# *Plasmodium yoelii *17XL infection up-regulates RANTES, CCR1, CCR3 and CCR5 expression, and induces ultrastructural changes in the cerebellum

**DOI:** 10.1186/1475-2875-4-63

**Published:** 2005-12-16

**Authors:** Bismark Y Sarfo, Henry B Armah, Ikovwaiza Irune, Andrew A Adjei, Christine S Olver, Shailesh Singh, James W Lillard, Jonathan K Stiles

**Affiliations:** 1 Parasitology Unit, Noguchi Memorial Institute for Medical Research, University of Ghana, P.O. Box LG581, Legon, Accra, Ghana; 2 Department of Microbiology, Biochemistry and Immunology, Morehouse School of Medicine, 720 Westview Drive S. W., Atlanta, GA, 30310-1495, USA; 3 Department of Pathology, University of Ghana Medical School & Korle-Bu Teaching Hospital, P.O. Box 4236, Accra, Ghana; 4 Department of Pathology, Colorado State University, Fort Collins, CO, 80523, USA

## Abstract

**Background:**

Malaria afflicts 300–500 million people causing over 1 million deaths globally per year. The immunopathogenesis of malaria is mediated partly by co mplex cellular and immunomodulator interactions involving co-regulators such as cytokines and adhesion molecules. However, the role of chemokines and their receptors in malaria immunopathology remains unclear. RANTES (Regulated on Activation Normal T-Cell Expressed and Secreted) is a chemokine involved in the generation of inflammatory infiltrates. Recent studies indicate that the degradation of cell-cell junctions, blood-brain barrier dysfunction, recruitment of leukocytes and *Plasmodium*-infected erythrocytes into and occlusion of microvessels relevant to malaria pathogenesis are associated with RANTES expression. Additionally, activated lymphocytes, platelets and endothelial cells release large quantities of RANTES, thus suggesting a unique role for RANTES in the generation and maintenance of the malaria-induced inflammatory response. The hypothesis of this study is that RANTES and its corresponding receptors (CCR1, CCR3 and CCR5) modulate malaria immunopathogenesis. A murine malaria model was utilized to evaluate the role of this chemokine and its receptors in malaria.

**Methods:**

The alterations in immunomodulator gene expression in brains of *Plasmodium yoelii *17XL-infected mice was analysed using cDNA microarray screening, followed by a temporal comparison of mRNA and protein expression of RANTES and its corresponding receptors by qRT-PCR and Western blot analysis, respectively. Plasma RANTES levels was determined by ELISA and ultrastructural studies of brain sections from infected and uninfected mice was conducted.

**Results:**

RANTES (p < 0.002), CCR1 (p < 0.036), CCR3 (p < 0.033), and CCR5 (p < 0.026) mRNA were significantly upregulated at peak parasitaemia and remained high thereafter in the experimental mouse model. RANTES protein in the brain of infected mice was upregulated (p < 0.034) compared with controls. RANTES plasma levels were significantly upregulated; two to three fold in infected mice compared with controls (p < 0.026). Some d istal microvascular endothelium in infected cerebellum appeared degraded, but remained intact in controls.

**Conclusion:**

The upregulation of RANTES, CCR1, CCR3, and CCR5 mRNA, and RANTES protein mediate inflammation and cellular degradation in the cerebellum during *P. yoelii *17XL malaria.

## Background

Malaria afflicts between 300–500 million people causing up to 2 million deaths globally per year [1]. Cerebral malaria (CM), characterized by seizures and loss of consciousness, is the most severe complication of *Plasmodium falciparum *infection with mortality rates ranging from 15 to 20% [2,3]. Malaria-induced brain inflammation is known to be mediated partly by complex cellular and immunomodulator interactions, involving co-regulators such as cytokines and adhesion molecules, resulting in the sequestration of parasite-infected erythrocytes in the brain in human CM. Apart from the sequestration of *P. falciparum*-infected erythrocytes, recent studies [4-7] have revealed significant accumulation of platelets and leukocytes in the distal microvasculature of the brains of human cases of CM, suggesting a role for platelet and leukocyte sequestration in human CM pathology. However, the role of chemokines and chemokine receptors in malaria brain immunopathogenesis still remain unclear. Recently, the up-regulated expression of RANTES and its receptors (C CR3 and CCR5) in the cerebellar and cerebral regions of post-mortem human CM brains has been reported [8]. Additionally, others [9,10] have reported increased migration of CCR5^+ ^leukocytes into the brain in experimental murine CM models. These studies support the hypothesis that leukocyte recruitment by chemokines may play a role in the pathogenesis of human CM. Indeed, malaria has become one of the many inflammatory diseases in which RANTES and its receptors appear to play a role. RANTES, a chemokine involved in the generation of inflammatory infiltrates, plays a special role in the maintenance and prolongation of the inflammatory response. The trafficking of inflammatory Th1 cells into the brain is mediated partly by RANTES interactions with CCR5. RANTES binds to a variety of receptors including CCR1, CCR3 and CCR5, expressed by monocytes/macrophages, memory T-cells, eosinophils, endothelial cells, basophils and mast cells [11]. A comparative study using *Plasmodium berghei *ANKA infected C57BL/6 and BALB/c mice indicated th at both strains of mice expressed CXCL10 (interferon-induced protein 10, IP-10) and CCL2 (monocyte chemotactic protein-1, MCP-1) chemokine genes as early as 24 hours post-infection [12]. Moreover, the expression of IP-10 and MCP-1 genes in KT5, an astrocyte cell line, was induced *in vitro *upon stimulation with a crude antigen of malaria parasites [12]. Other more recent studies, using malaria animal models, showed that experimental cerebral malaria (ECM) was induced in perforin-deficient mice (PFP^-/-^) after adoptive transfer of cytotoxic CD8^+ ^T cells from infected C57BL/6 mice, which were directed to the brains of PFP^-/-^mice. This specific recruitment involved chemokines and their receptors, and indicated that lymphocyte cytotoxicity and trafficking are key players in ECM [10]. While CCR2 was not observed to be essential for the development of ECM [13], CCR5 deficiency in mice reportedly decreased susceptibility to ECM [9]. These studies, together, support the hypothesis that leukocyte recruitment by chemokine and chemokine receptor interactions play a role in the pathogenesis of malaria in these animal models. It seems that plasmodial infection has a significant impact on brain endothelial and parenchymal cells and, thus, provides a new dimension to our understand ing of the role of systemic and localized (brain) chemokine expression in CM immunopathogenesis. The cytoadherence of infected red blood cells (IRBCs) to the postcapillary venules is the major cause of IRBC sequestration and vessel blockage in the cerebral form of human malaria. In both human cerebral malaria caused by *P. falciparum *and the *Plasmodium yoelii *17XL-infected rodent model of malaria, the sequestration of IRBCs in the brain vessels is secondary to the cytoadherence of IRBCs to the postcapillary venules [14]. In this study, we analysed the alterations in immunomodulator gene expression in brain samples of *P. yoelii *17XL-infected mice using cDNA microarray screening, coupled with analysis of temporal expression patterns of RANTES and its corresponding receptors, CCR1, CCR3 and CCR5, in brain samples and plasma of *P. yoelii *17XL-infected mice to identify and characterize the role of these immunomodulators during rodent malaria.

## Methods

### Murine model of *P. yoelii *17XL malaria and preparation of brain samples

All animal-related experiments were conducted according to the principles set forth in the *National Institutes of Health Guide for the Care and Use of Laboratory Animals *and approved by the Institutional Animal Care and Use Committee (IACUC) of Morehouse School of Medicine. Female Swiss Webster (SW) mice (6–8 weeks) obtained from Jackson Laboratory (Bar Harbour, Maine, USA) were maintained on a 12 hr light/dark cycle with access to food and water *ad libitum*, in accordance with IACUC regulations of the Association for Assessment and Accreditation of Laboratory Animal Care (AAALAC). Humane methods were used to sedate mice prior to intra-peritoneal injection and tail-snipping. Briefly, the inhaled anesthetic (0.1–0.2 ml isoflurane or halothane per liter of induction chamber volume to give a gas concen tration of 2–4%, required to induce anesthesia) were administered to the female SW mice. The induction process was visualized through the anesthetic chamber and the abolition of the toe-pinch pain reflex assessed by applying pressure to indicate the successful induction of anesthesia. Mice were injected intraperitoneally with *P. yoelii *17XL parasitized blood, kindly provided by Dr. Christine Olver (Department of Pathology, Colorado State University, USA). This rodent malaria strain causes a syndrome that resembles human malaria, char acterized by fever, spleno- and hepatomegaly by day eight post-infection [14,15]. Parasitaemia was determined in a total count of 300 to 500 red blood cells (RBCs) on Wright-Giemsa-stained (Sigma Diagnostics, USA) thin blood smears. Euthanasia was conducted by the inhalation of CO2 or cervical dislocation, and groups of fifteen infected and uninfected mice were sacrificed after day two, four, six and eight post-infection. For each time point, five brains from infected mice were stored in RNA later (AmbionTM Inc., USA) at -80°C for RNA isolation, five brains were stored in Lysis Buffer at -80°C for protein analysis, and 5 were cryoprotected in 4% paraformaldehyde at 4°C for light and transmission electron microscopy. Similarly, brains from uninfected mice were stored for RNA isolation, protein analysis, and light and transmission electron microscopy.

### RNA isolation

Messenger RNA (mRNA) was isolated from brain samples using TRIzol Reagent (Life Technologies Inc., Rockville, MD., USA) according to the manufacturer's protocol. Genomic DNA contamination was removed from these samples by treatment with RNase-free DNase (Invitrogen, San Diego, CA, USA) for 15 minutes at 37°C. RNA was then precipitated and re-suspended in diethylpyrocarbonated (DEPC)-treated water.

### cDNA microarray screening

Five micrograms (5 μg) of DNA-free RNA from day 8 post-infected (peak parasitaemia) mouse brain was reverse transcribed, in the presence of 100 μCi of [alpha-^32^P] dATP, for microarray analysis. The commercial system used to investigate cDNA microarray gene expression (AtlasTM 1.0; CLONTECH, Palo Alto, CA., USA) consists of two identical nylon membranes, spotted with 588 different mouse genes grouped in functional blocks, including immunomodulators, neurotrophins, neurotransmitters and pro- and anti-apoptosis genes. A complete list of the cDNA samples and controls on each array, as well as their corresponding GenBank accession numbers, may be found at CLONTECH's Atlas web site . Briefly, [alpha-^32^P] dATP-labelled cDNA synthesized from the 5 μg DNA-free RNA by reverse transcription was column purified and hybridized, at high stringency, to a mouse cDNA array overnight at 70°C. Membranes were washed at high stringency and exposed to X-ray film at -80°C overnight, as recommended by the manufacturers. Message expression was analysed using the AtlasTM Image 1.0 software (CLONTECH, Palo Alto, CA., USA) and the data expressed as the ratios of the relative changes in the mRNA levels in the infected brain samples and uninfected controls. Expression of mRNA populations (from infected and uninfected mice) at day 8 post-infection were compared and analysed by autoradiography and quantified by CLONTECH gene an alysis software. An approximate estimate of the abundance level of a target cDNA in an RNA population can be made by comparing its signal to the signals obtained with housekeeping genes of known abundance (e.g. GAPDH).

### RT-PCR validation of immunomodulator mRNA expression

Genes encoding induced RANTES, CCR1, CCR3 and CCR5 were selectively confirmed by semi-quantitative RT-PCR. Mouse mRNA sequences of RANTES, CCR1, CCR3, CCR5 and glyceraldehyde-3-phosphate dehydrogenase (GAPDH) were obtained from the National Institute of Health -National Center for Biotechnology Information (NIH-NCBI) GeneBank database accession numbers [AF_252285], [NM_009912], [NM_009914], [D_83648] and [NM_008084] respectively. These sequences were then used to design primers for RT-PCR analysis, which generated amplicons of 97, 103, 96, 100, and 95 base pairs in sizes for RANTES, CCR1, CCR3, CCR5 and GAPDH mRNA respectively. Primers were designed using the primer 3 software program from the Whitehead Institute at the Massachusetts Institute of Technology (MIT; Boston, MA., USA). Thermodynamic analysis of the primers was conducted using computer programs: Primer Premier TM (Integrated DNA Technologies, Coralville, Iowa, USA), and MIT Primer III (Boston, MA., USA). The resulting primer sets were compared against the entire mouse genome using NCBI to confirm specificity and ensure that the primers flanked mRNA splicing regions. Complementary DNA (cDNA) was generated (Maxim Biotech Inc., CA., USA), and amplified with specific primers using *Taq *polymerase and polymerase chain reaction (PCR) reagents (Qiagen Inc. Valencia, CA., USA). The levels of band intensities of mRNA of these targets relative to GAPDH were evaluated by PCR analysis using thermocycler (Perkin-Elmer, Norwalk, Conn., USA). Conditions for DNA amplifications were set as follows: heating at 94°C for five minutes, followed by 25 cycles of DNA denaturation at 94°C for one minute, an annealing step at X°C (see Table 1 for respective temperature values for each primer) for one minute, strand exten sion at 72°C for one minute and a final extension step at 72°C for 10 minutes. The number of cycles required to attain products in the linear range of the PCR was determined before the final assay was run. Working within this range, it was possible to determine expression differences after 25–30 cycles. PCR products were analysed on 2%-agarose/ethidium-bromide gels and quantified using Gelexpert software (NucleoTech, San Mateo, CA, USA). Band intensities in each experiment were normalized to the mean intensity of GAPDH. Data were expressed as the relative change in mRNA level in infected and uninfected controls.

**Table 1:** Primer sequence with corresponding annealing temperatures

Mouse Gene	GenBank Accession Number	Sequence (Forward)	Sequence (Reverse)	Annealing Temp (°C)
RANTES	AF252285	GGAAATCTTCGCACCTCAAG	GAGCGTGCGAACTTCTTGTT	55
CCR1	NM009912	CCACTCCATGCCAAAAGACT	ATTAGGACATTGCCCACCAC	50
CCR3	NM009914	TATCATTACCTGGGGCCTTG	CGAGGACTGCAGGAAAACTC	53
CCR5	D83648	CGAAAACACATGGTCAAACG	GTTCTCCTGTGGATCGGGTA	52
GAPDH	NM_008084	CACAATTTCATCCCAGACC	GTGGGTGCAGCGAACTTTAT	55

### Western blot analysis of RANTES

Western blot analysis was used to confirm RANTES protein expression in mouse brain during *P. yoelii *17XL infection. Infected and uninfected mice brains were lysed and their total protein determined by standard methods using a commercial kit (BioRad, Hercules, CA, USA). Twenty five micrograms (25 μg) of total protein from mouse brain was subjected to 15% SDS-PAGE, and blo tted onto nitrocellulose membranes. Membranes were then probed with 1:1000 biotinylated anti-mouse recombinant RANTES (R&D Systems, Inc. MN, USA) and 1:2000 anti-mouse alpha-tubulin (Sigma-Aldrich, MO, USA) antibody. Membranes probed with biotinylated anti-mouse recombinant RANTES were incubated with streptavidin-horseradish peroxidase (HRP) secondary antibody while those probed with alpha-tubulin were incubated with anti-mouse IgG secondary antibody at the same conditions. The methods used for pre-incubatio n, incubation and detection by chemiluminescence have already been described [16]. Bands of protein corresponding to RANT ES (7.8 kDa) and alpha-tubulin (55 kDa) were quantified using Versa Doc Imaging System (BioRad, CA, USA). RANTES protein expression was normalized to that of alpha-tubulin.

### ELISA determination of plasma RANTES levels

To determine whether RANTES and its receptor interactions were localized (brain) or systemic (peripheral blood), plasma RANTES levels were determined in *P. yoelii *17XL-infected and control mice, using RANTES specific ELISA (Biosource International, Camarillo, CA, USA) according to the manufacturer's specifications. Since RANTES may be released by platelets during serum collection, heparinized blood was collected, centrifuged at 13,000 rpm for 10 minutes to obtain plasma samples, and subsequently stored at -20°C until used. Briefly, duplicates of standard controls, and samples were aliquoted into RANTES-coated microtiter wells. Biotin-conjugated antibody was added to the wells and incubated at room temperature for 2 hours. Streptavidin-horseradish peroxidase (Streptavidin -HRP) was then added to each well and incubated at room temperature for 30 min. The plates were developed with stabilized chromogen in the dark at room temperature. The reaction was stopped and optical densities of samples were read at 450 nm.

### Histopathologic analysis of *P. yoelii *17XL infected mouse brain

Whole brains of infected and uninfected mice at peak parasitaemia were examined by light microscopy to evaluate erythrocytic and leucocytic sequestration in brain microvasculature. Whole mouse brains fixed in 4% paraformaldehyde were processed for routine histology, with haematoxylin and eosin staining. Sections of these paraformaldehyde-fixed samples (20 randomly selected sections of microvessels) from each brain were scored positive or negative for erythrocyte and leukocyte sequestration. The percentages of microvessels from each brain that showed erythrocytic and leucocytic sequestration were noted.

### Ultrastructural analysis of *P. yoelii *17XL infected mouse brain

Whole brains of infected and uninfected mice at peak parasitaemia were examined by electron microscopy to evaluate parasite induced morphological changes in brain microvasculature. Brains were dissected, cut into smaller cubes (2 mm^3^), washed twice with phosphate buffered saline (PBS) and fixed for 60 minutes in 100 mM potassium phosphate buffer pH 7.2, containing 0.1 % gluteraldehyde and 2% freshly prepared formaldehyde. After fixation the brains were dehydrated in methanol and embedded in Lowicryl K4M at -20°C. Thin sections were collected on 300-mesh nickel grids and examined by transmission electron microscopy at 60 eV as described previously [17].

### Statistical analysis

The results obtained in this work were from triplicate experiments performed independently by identical methods. ELISA data and densitometric measurements from agarose gel electrophoresis, as well as Western blot analyses, were log-transformed to normalize the distribution for infected (n = 15) and control (n = 15) samples, and also to correct for small sample size. Data were expressed as the mean ± standard error of mean (SEM). Data from the *P. yoelii *17XL infected and control groups were then compared, and the p values were determined by using nonparametric Mann-Whitney U-test. A value of p < 0.05 was considered statistically significant.

## Results

### *Plasmodium yoelii *17XL murine malaria

**Figure 1:**
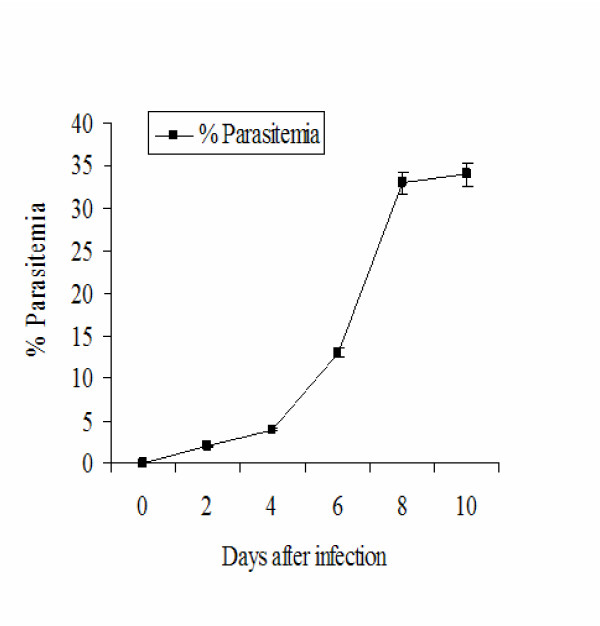
**Course of *P. yoelii 17XL *infection in female SW mice. **Level of parasitaemia in P. yoelii 17XL infected mice was monitored from the tail vein blood and counting at least 300 RBCs under immersion oil. Data represent mean ± SEM of 5–10 counts per point.

All the mice infected with *P. yoelii *17XL parasites developed malaria-related-symptoms, which included the appearance of ruffled fur and shivering at peak parasitaemia by day eight post-infection (Figure 1). Examination of the viscera of dissected mice confirmed spleno- and hepatomegaly at peak parasitaemia, concordant with reported *P. yoelii *17XL malaria infections [14,15]. None of the control or uninfected mice showed any of these signs. T he mice infected with *P. yoelii *17XL did not develop the classic signs of cerebral pathology (such as hemiplegia, paraplegia, ataxia with hind limb paralysis, convulsions and coma associated with murine CM previously described in the *Plasmodium berghei *ANKA murine CM model [18,19]). Additionally, histopathologic analysis of brains of the *P. yoelii *17XL-infected mice revealed plugging of brain microvessels with parasitized erythrocytes, but d id not reveal evidence of disseminated petechial haemorrhages and extensive leukocyte accumulation in the microcirculation. However, there was ultrastructural evidence of oedema and disintegrating microvascular endothelia in the cerebellum, which reflect local perturbations induced by the *P. yoelii *17XL infection.

### cDNA microarray screening

**Figure 2:**
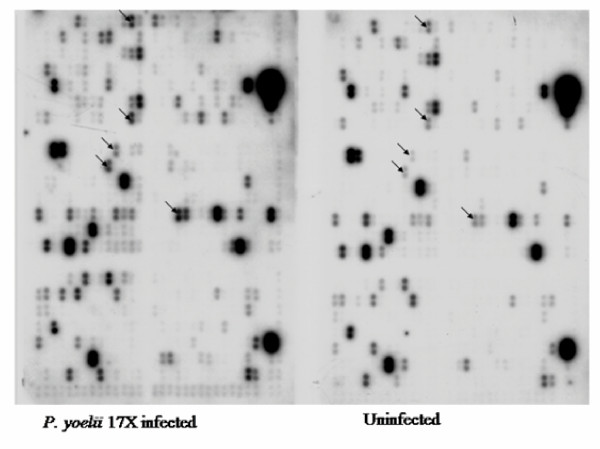
**Autoradiograph of cDNA microarray of *P. yoelii *17XL infected and uninfected control mice brains. **Arrows indicate some differences in intensity in expression of im munomodulator gene expression between *P. yoelii *17XL infected and control mice brains.

*P. yoelii *17XL-attributable alterations in approximately 7.5% (44/588) of genes encoding immunomodulators, growth factors, stress factors, transcription factors and neurotransmitters were observed in infected mouse brain during the microarray analysis. Expression of the altered genes at peak parasitaemia in the infected mice varied when compared with that in the uninfected mice (Figure 2). Marked alterations in expressio n of immunomodulator mRNA, including C-C chemokine RANTES, C-C chemokine receptors CCR1, CCR3 and CCR5, adhesion molecules PECAM-1, ICAM-1, and VCAM-1, cytokines IF N-gamma, TNF-alpha, IL-12, IL-4, and iNOS were observed to be up-regulated, while growth factors, GDF-2 and TGF-beta precursor, were down-regulated (Table 2, P < 0.05) at peak parasitaemia.

**Table 2:** cDNA microarray analysis of immunomodulator gene expression in *P. yoelii *17XL infected mouse brain at peak parasitemia (day eight post-infection).

**GENE NAME**	**FOLD CHANGE EXPRESSION**	**DESCRIPTION OF GENE**
PECAM-1	23.0	Platelet Endothelia Cell Adhesion Molecule-1
ICAM-1	13.0	Intercellular Adhesion Molecule-1
VCAM-1	6.0	Vascular Cell Adhesion Molecule-1
**RANTES**	**6.0**	**Regulated upon Activation Normal T cell Expressed and Secreted**
INF-γ	6.0	Interferon-gamma
TNF	5.0	Tumor Necrosis Factor-alpha
IL-12	4.0	Interleukin-12
**CCR1**	**4.0**	**C-C chemokine receptor 1**
**CCR3**	**4.0**	**C-C chemokine receptor 3**
**CCR5**	**4.0**	**C-C chemokine receptor 5**
iNOS	4.0	Inducible Nitric Oxide Synthase
ZO-1	2.0	Zonula Occludin -1
IL-4	2.0	Interleukin-4
GDF-2	-2.0	Growth differentiation factor-2
TGF-α	-2.0	Tumor growth factor-alpha
**GAPDH**	**0.0**	**Glyceraldehyde-3-Phosphate Dehydrogenase**

*P. yoelii *17XL infection alters immunomudulator gene expression in mouse brain at peak parasitemia (day eight post-infection). cDNA microarray analysis of *P. yoelii *17XL infected and uninfected mouse brain. Gene listed were altered > 1.5 fold at peak parasitemia. Fold change in expression is defined as GAPDH normalized mRNA expression ratio of gene signals of infected to uninfected mouse brain.

### RT-PCR validation of immunomodulator mRNA expression

**Figure 3:**
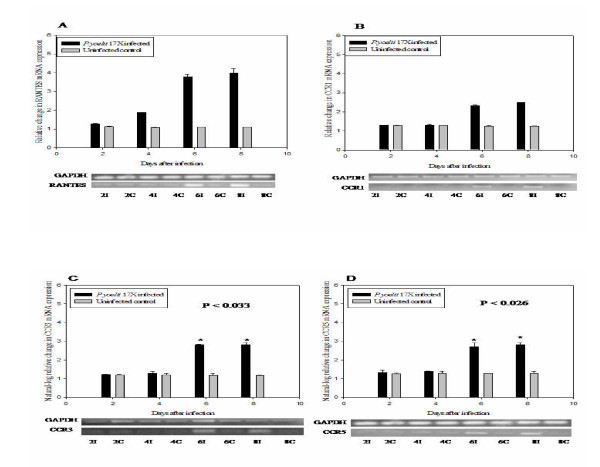
***P. yoelii *17XL upregulates RANTES, CCR1, CCR3 and CCR5 mRNA expression in mouse brain. **Semi-quantitative RT-PCR comparative analysis of RANTES CCR1, CCR3 and CCR5 mRNA expressions in brains of *P. yoelii *17XL-infected (I, black bars) days two, four, six, and eight post-infections and control (C, grey bars) mice. Densitometric data presented were log-transformed (to adjust for small sample size) and represent the mean and ± SEM of infected (n = 5) and control (n = 5) cases, normalized to those for GAPDH. Asterisk(s) indicate statistically significant differences between *P. yoelii *17XL infected and control groups.

The expression of RANTES and its corresponding receptors, CCR1, CCR3 and CCR 5, were altered by *P. yoelii *17XL infection and were significantly up-regulated (p < 0.002 for RANTES, p < 0.036 for CCR1, p < 0.033 for CCR3 and p < 0.026 for CCR5) in the brain during malaria infection. Up-regulation of RANTES mRNA began four days after infection, until eight days post-infectio n, approximately three-fold increase in infected mouse brain at day six and day eight post-infection compared with controls [Figure 3A]. Messenger RNA expression levels of CCR1, CCR3 and CCR5 were approximately two to three fold higher in infected mice than in controls [Figures 3B, 3C &3D]. CCR3 and CCR5 expression profiles were similar to the expression profile of their corresponding RANTES ligand. The degree of variation of GAPDH mRNA from sample to sample was within 5% of the mean expression level in both infected and uninfec ted control samples throughout the course of the infection.

### Western blot analysis

**Figure 4:**
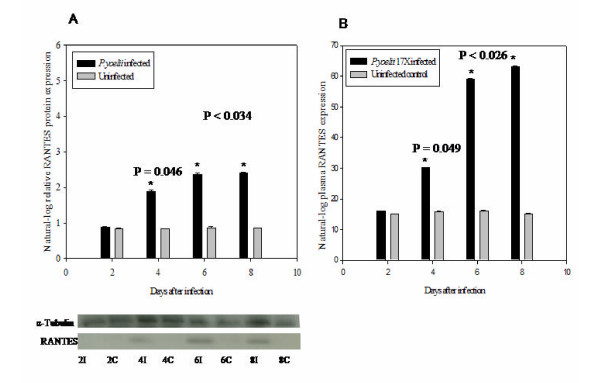
***P. yoelii *17XL infection upregulates RANTES protein expression in plasma and mouse brain. **Comparative analysis of RANTES (7.8 kDa) protein expression in brain tissue samples (A) and plasma (B) from *P. yoelii *17XL-infected mice days two, four, six, & eight post-infection (black bars) versus uninfected controls (grey bars). Brain tissue protein and plasma samples from infected and uninfected mice were analysed for RANTES expression by Western Blot and ELISA respectively. Densitometric data of Western blots were log-transformed (to adjust for small sample size) and represent the mean and ± SEM of infected (n = 5) and control (n = 5) cases, normalized to those for atubulin. Asterisk(s) indicate statistically significant differences between *P. yoelii *17XL-infected and control groups.

Results from the Western blots analysis indicated that the expression of RANTES (7.8 kDa) protein in brain tissue samples from infected mice at day four, six and eight post-infection, was significantly up-regulated (p = 0.049 at day four and p < 0.036 at day six and day eight) [Figure 4A]. Expression of RANTES protein in brain tissues followed a similar profile consistent with mRNA expression indicating that RANTES mRNA expression in brains of *P. yoelii *17XL infected mice were translated into protein. Expression of alpha-tubulin protein from sample to sample was within 5% of the mean expression level in both infected and uninfected samples throughout the course of the infection.

### Plasma RANTES protein level during *P. yoelii *17XL infection

Plasma samples from infected mice at each time point as well as uninfected controls were assayed for RANTES protein expression. Systemic increase in RANTES protein expression began four days after *P. yoelii *17XL infection, until peak parasitaemia at day eight with about three-fold upregulation in infected plasma compared with control [Figure 4B].

### Histopathologic analysis of mouse brain

Sequestered parasitized erythrocytes were observed in brain microvessels of all the *P. yoelii *17XL-infected mice studied at peak parasitaemia, but none of the uninfected mice. There was no histopathologic evidence of sequestered or accumulated mononuclear leukocytes (monocytes/macrophages and lymphocytes) or extensive petechial haemorrhages in the brains of both the infected and uninfected mice. In the brain of the infected mice, the erythrocyte sequestration observed in the white and grey matter regions was identical.

### Ultrastructural analysis of mouse brain

**Figure 5:**
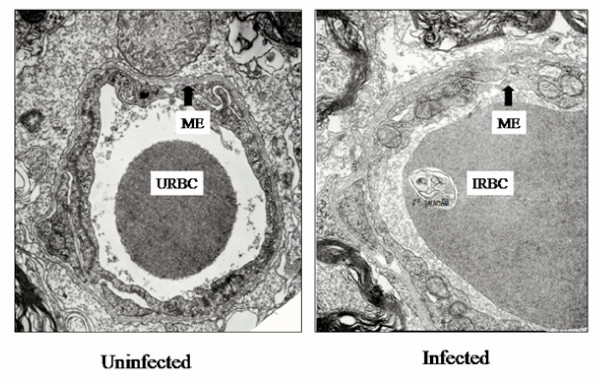
***P. yoelii *17XL induces ultra structural changes in mouse cerebellum at peak parasitaemia (day eight post-infection) [scale bar: 1 μm]. **Cerebellum from parasitized mice was ultra structurally analysed by transmission electron microscopy to evaluate effects of *P. yoelii *17XL infection on brain micro vessels. Uninfected mice (Mag. × 15000) show normal intact micro vessel endothelia (ME) at blood brain barrier (BBB). Infected mice (six out of 10) [Mag. × 15000] show disintegrating ME and BBB (arrow) of brain vessels examined at day eight post-infection. Erythrocytic (RBC) adherence in the microvessels, but not leukocytic (WBC) adherence, was observed in the infected mouse brains examined.

Brains from parasitized mice were ultrastructurally analysed by transmission electron microscopy to evaluate effects of *P. yoelii *17XL infection on brain microvessel endothelium. Uninfected mouse cerebellar tissues (Mag. × 15000) showed normal intact microvessel endothelia (ME) at blood b rain barrier (BBB). Infected mouse cerebellar tissues (Mag. × 15000) showed peri-vascular clearing concomitant with oedema as well as some disintegrating ME and BBB [Figure 5]. This endothelial cell damage (lesions) was observed in 6 out of 10 mice examined at day eight post-infection. Erythrocytic (RBC) adherence in the microvessels, but not leukocytic (WBC) adherence, was observed in the infected mouse brains examined.

## Discussion

The brain pathology associated with malaria remains a major cause of death d uring severe *P. falciparum *infection. Cerebral malaria, characterized by coma and seizures in patients with *P. falciparum *infection, is a major cause of malaria associated mortality, and may be accompanied by metabolic acidosis and hypoglycaemia in African children [20]. Using experimental models will facilitate a better understanding of the pathogenesis of this syndrome and therefore ensuring that better intervention strategies can be developed to minimize or abrogate the severity of the disease. The cytoadherence of infected red blood cells (IRBCs) to the postcapillary venules is the major cause of IRBC sequestration and vessel blockage in the cerebral form of human malaria. In both human cerebral malaria caused by *P. falciparum *and the *P. yoelii *17XL-infected rodent model of malaria, the sequestration of IRBCs in the brain vessels is secondary to the cytoadherence of IRBCs to the postcapillary venules [14,15]. This observation has resulted in the general suggestion that the *P. yoelii *17XL mouse model resembles human *P. falciparum *infection more closely than the *P. berghei *ANKA mouse model, since it shows little accumulation of monocytes /macrophages in the brain microvessels [14,15]. However, recent human CM studies [4-7], indicating significant ac cumulation of platelets and leukocytes in the distal cerebral microvasculature in CM, suggest some other similarities between human CM and the *P. berghei *ANKA mouse CM model, in addition to the similarities in symptomatology [18,19]. These recent reports [4-7] of significant leukocyte accumulations in the brain microvasculature in human CM draws a similarity with the *P. berghei *ANKA-infected rodent model of malaria, in which the major histopathologic finding is extensive accumulation of monocytes or macrophages, rather than sequestered erythrocytes, in the brain [18,19].

In this study, all the mice infected with *P. yoelii *17XL developed malaria-related symptoms, which included the appearance of ruffled fur and shivering by peak parasitaemia at day eight post-infection. Spleno- and hepato-megaly at peak parasitaemia was common, and concordant with reported *P. yoelii *17XL malaria infections [14,15]. The observation of the absence of the classic signs of cerebral pathology in the *P. yoelii *17XL-infected mice at peak parasitaemia and the histopathologic findings of IRBC sequestration and vessel plugging with the absence of leucocyte accumulation in brains of *P. yoelii *17XL-infected mice, confirms previously reported observations [14,15]. The classic signs of cerebral pathology, namely hemiplegia, paraplegia, ataxia with hind-limb paralysis, convulsions and coma, have been previously described in the *P. berghei *ANKA mouse model [18,19]. These observations provide a justification for the complementary use of both murine malaria models to study human CM. The *P. berghei *ANKA model shows similarity with human CM in terms of symptomatology, whilst the *P. yoelii *17XL model exhibits similarity to human CM in terms of histopathology. This study focused mainly on malaria induced chemokine and chemokine receptor expression in the *P. yoelii *17XL murine model. Animal models have provided compelling evidence implicating the role of inflammatory processes in the development of malaria brain pathology [21]. Adhesion molecules and platelet-induced immune-mediated damage of vascular endothelium of the brain have also been reported [21]. Tkachuk and colleagues observed that malaria infection induced the expression of CCR3 and CCR5 on placental macrophages in pregnant women [22]. Sarfo and colleagues indicated that RANTES and its receptors CCR3 and CCR5 were upregulated in the cerebellum and cerebrum of post-mortem human CM tissue samples [8]. Furthermore, activated T-lymphocytes, platelets and endothelial cells release large amount of RANTES 3–5 days after activation, giving this chemokine a unique role in the generation, maintenance and prolongation of immune and inflammatory response [23]. By understanding the role of RANTES and its receptors during malaria immunopathogenesis, a new strategy for preventing or minimizing the outcome of CM and other severe forms of malaria can be developed. The microarray results (Table 2) confirmed with semi-quantitative RT-PCR analysis from this study revealed changes in the expression of a number of immunomodulators that had previo usly been associated with malaria-induced brain dysfunction [12]. In this study, the expressions of RANTES and its corresponding receptors CCR1, CCR3 and CCR 5 were up-regulated in the brain during *P. yoelii *17XL infection, further implicating these molecules in the pathogenesis of rodent cerebral malaria. This study is a first towards the development of a molecular fingerprint (diagnostic) for brain immunopathogenesis associated with malaria. In this regard, recent studies with infectious agents such as *Salmonella*, *Chlamydia *and *Trypanosoma *using cDNA microarray technology have revealed unique gene-expression profiles [24-26] which may be of diagnostic value.

This study demonstrates that chemokine RANTES (CCL5) and its receptors (CCR1, CCR3 and CCR5) may play an important role in *P. yoelii *17XL infection in mice. Chemokines are immunoregulatory factors that play an important role in the chemotaxis, activation and haematopoiesis of leukocytes [27-29]. Chemokine action involves initial binding to specific, seven-transmembrane-domain, G-(guanine-nucleotide-binding)-protein-coupled receptors on target cells. In response to a relatively higher concentration of chemokines at the site of injury or infection, leukocytes are activated to perform effector functions such as release of their granule contents and increased production of cytokines. The temporal expression profile of chemokines and their receptors as early immunomodulators in the immunopathogenesis of malaria could serve as important new bio-markers for monitoring the course and predicting the outcome of the disease. The cDNA microarray analysis has revealed significant up-regulation of RANTES (6fold) at peak parasitaemia. The results of RT-PCR analysis indicate that by days 6 through 8 post-infection, mRNA expression of RANTES is significantly up-regulated (p < 0.002) in infected mice compared with controls, indicating that it is involved in the immunopathogenesis in *P. yoelii *17XL-infected mouse. RANTES in addition to CCR1 and CCR5 are expressed by Th1 cells [30]. Indeed, trafficking of inflammatory Th1 cells into the brain was reportedly mediated largely by RANTES interaction with CCR5 receptor [30]. Also the absence of CCR5 receptor in *Plasmodium berghei *ANKA-infected mouse brain resulted in a reduced Th1 cytokine production [9]. The expression of RANTES and CCR5 mRNA in *P. yoelii *17XL-infected mouse brain in this study suggests a Th1-mediated immune response, and that factors capable of inducing Th1 response could play an important role in modulating malaria infections. Macrophages and other leukocytes release proinflammatory cytokines, including TNF-alpha, IFN-gamma and IL-1-beta, which in turn will promote the release of chemokines [30]. The expression of chemokine, IP-10 and MCP-1, genes in KT-5, an astrocyte cell line, have been shown to be upregulated in vitro upon stimulation with a crude antigen of malaria parasites [12]. A soluble gradient of these chemokines within the tissue recruits various cell types that express receptors for the different chemokines. The expressions of all the C-C chemokine receptors for RANTES, CCR1, CCR3 and CCR5, were upregulated in the brains of *P. yoelii *17XL-infected mice. The expression of RANTES probably enhanced the expression of its receptors. Sano and colleagues demonstrated that ICAM-1 induced RANTES mRNA expression and also increased its protein synthesis and secretion by endothelial cells [31]. It is likely that the *P. yoelii *17XL-induced RANTES production observed in the current study would attract and activate leukocytes towards inflammatory sites to mediate localized hyper-inflammatory responses that could exacerbate the disease pathology in the cerebellum. Belnoue and colleagues showed that the brains of wild -type mice with CM have significantly higher levels of CCR5 than the knockout-type, implicating these molecules in the pathological changes produced in the brain during the infection [9]. The results of this study demonstrate that the increase in production of RANTES follows the course of *P. yoelii *17XL malaria infection, thus RANTES and its receptors CCR1, CCR3 and CCR5 were detected at their highest levels at day six and day eight post-infection. This observed temporal association of the progression of *P. yoelii *17XL infection with the increasing production of RANTES and its receptors suggests that the two events might be linked. Western blot analysis revealed that brain tissue transcripts of RANTES were actually translated into protein, and were significantly up-regulated (p = 0.046 for day 4 and p < 0.034 for day six and day eight post-infection) in infected mice (Figure 3A). The ELISA data from this study indicate significant upregulation (p = 0.049 for day 4 and p < 0.026 for day six and day eight post-infection) of RANTES in *P. yoelii *17XL-infected mouse plasma than in controls (Figure 3B). Most of the ultrastructural pathological changes, observed as endothelial cell damage (lesions) in six out of the 10 infected mice examined, occurred especially at day eight post-infection (peak parasitaemia) [Figure 5]. Thus, the increase in RANTES production correlated with the increase in parasitaemia and pathological changes observed in the *P. yoelii *17XL-infected mice in this investigation. Chemokines have been shown to have a direct antiprotozoal activity for three protozoans: *Toxoplasma gondii, Leishmania donovani *and *Trypanosoma cruzi *[32,33]. Chemokine production is important for defending the host against infection. However, excessive production is also deleterious to the host [12]. It has been observed that C-C chemokines, such as MIP-1-alpha and RANTES, are significantly upregulated in brains of *Trypanosoma brucei brucei *infected rats [34]. This increase in expression of these chemokines occurs before brain lesions developed in infected rats, implying that the induction of these chemokines could be directly responsible for the observed rat brain lesions [34]. Ultrastructural analysis of mouse brain by electron microscopy at peak parasitaemia in this study, revealed disintegrating microvascular endothelial layer at the blood brain barrier in the cerebellar region of infected mouse brain. This endothelia l cell damage (lesions), in six out of 10 mice examined, occurred especially at day eight post-infection (peak parasitaemia) and was similar to the observations of Thumwood and colleagues in *P. berghei *ANKA-infected A/J and CBA/H mice [35]. The infected erythrocytes adhering and occluding brain microvessels observed in the sections examined, suggest that the breach in the cerebellar microvascular endothelial layer could be associated with parasite-induced inflammation or apoptosis. Perivascular oedema was also observed in this region of infected mouse brain probably as a result of the endothelial cell damage allowing excess fluid to move across the blood brain barrier. End othelial cells interacting with *P. yoelii *17XL-parasitized erythrocytes have been shown to be induced to produce and present specific chemokines, such as RANTES, which can lure CCR1, CCR3 and CCR5 expressing cells into the brain [36]. CCR1 and CCR 5 are exp ressed by brain endothelial cells [37,38]. Brain endothelial cells, microglia and astrocytes, which are the 3 major cellular components of the BBB, express CCR5 receptor [39], and hence the binding of RANTES to its receptors on these cells can serve to further activate them and enhance a localized breakdown of the microvessel endothelial layer observed in the infected mouse brain in the current investigation.

## Conclusion

In conclusion, *P. yoelii *17XL infection upregulates RANTES and its corresponding receptors, CCR1, CCR3 and CCR5, in mouse brain, and that ultrastructural changes in the microvascular endothelial layer occurred in the cerebellum of infected mice. This is the first temporal expression study of RANTES and its receptors associated with murine malaria. Further studies are underway to examine the expression of these chemokines and chemokine receptors in a human CM symptomatology-like model such as *P. berghei *ANKA, to ascertain differences and similarities. As it is not clear which cell types in the mouse CM brain samples over-express RANTES, CCR1, CCR3 and CCR5, further comparative immunolocalization and antibody ablation studies are currently underway to examine the physiological relevance, source and expression patterns of these important biomarkers in both murine and human CM brain samples.

## List of abbreviations used

BBB, Blood-Brain Barrier;

CM, cerebral malaria;

ECM, experimental cerebral malaria;

ME, microvascular endothelium;

NCM, non-cerebral malaria;

*P., Plasmodium*;

RBC, red blood cell;

WBC, white blood cell;

TNF, Tumor Necrosis Factor;

IFN, Interferon; IL, Interleukin

## Authors' contributions

BYS, HA, II and AAA carried out the animal manipulations, molecular genetic studies, Western blots, immunoassays, electron microscopy, data analysis, and participated in the sequence alignments and participated in drafting of the manuscript. CSO provided characterized parasite strains (*P. yoelii *17XL) and murine malaria model. SS and JWL participated in the design of the study, provided chemokine antibodies and oligonucleotide primers for Western blots and RT-PCR, respectively. JKS conceived of the study, participated in its design and coordination and helped to draft the manuscript. All authors read and approved the final manuscript.
